# A mixed methods study to investigate needs assessment for knee pain and disability: population and individual perspectives

**DOI:** 10.1186/1471-2474-8-59

**Published:** 2007-07-04

**Authors:** Clare Jinks, Bie Nio Ong, Jane Richardson

**Affiliations:** 1 Primary Care Musculoskeletal Research Centre, Keele University, Keele, ST5 5BG, UK

## Abstract

**Background:**

The new Musculoskeletal Services Framework outlines the importance of health care needs assessment. Our aim was to provide a model for this for knee pain and disability, describing felt need (individual assessment of a need for health care) and expressed need (demand for health care). This intelligence is required by health care planners in order to implement the new Framework.

**Methods:**

A multi-method approach was used. A population survey (n = 5784) was administered to adults aged 50+ registered with 3 general practices. The questionnaire contained a Knee Pain Screening Tool to identify the prevalence of knee pain and health care use in the population, and the Western Ontario and McMaster Universities Osteoarthritis Index (WOMAC). Survey responders who scored "severe" or "extreme" on at least one item on the pain or physical function scale on the WOMAC were categorised into "severe" groups. Qualitative interviews were undertaken with 22 survey responders to explore in detail the experience of living with knee pain and disability. A sample of interviewees (n = 10) completed an open format patient diary to explore the experience of knee pain in everyday life.

**Results:**

The 12-month period prevalence of knee pain was 49.5%, of which half was severe. Severe difficulties were reported with domestic duties, bending, bathing, climbing stairs and getting in or out of a car. Some self-care is occurring. The majority (53%) of responders with severe pain or disability had not consulted their GP in the last 12 months. The qualitative study revealed reasons for this including a perception that knee pain is part of normal ageing, little effective prevention and treatment is available and the use of medications causes side effects and dependency.

**Conclusion:**

This study adds to previous work by highlighting a gap between felt and expressed need and the reasons for this mismatch. There is evidence of self-management, but also missed opportunities for effective interventions (e.g. lifestyle advice). A targeted and integrated approach between clinicians and health care planners for primary and secondary prevention is required if aspects of the new Musculoskeletal Services Framework are to be successfully implemented.

## Background

Understanding the health care needs of people with musculoskeletal conditions is now recognised in UK health care policy through the Musculoskeletal Services Framework (MSF) [1]. Previous needs assessments in this area have tended to focus on the needs for total joint replacement [2]. The new framework, however, describes needs assessment in a broader context in order to understand the prevalence and incidence of musculoskeletal disorders and identify where patients are and their use of services. This investigation is central to the basic delivery cycle of the NHS which incorporates population based needs assessment, identifying priorities and standards, planning services, commissioning services to meet needs and assessing outcomes [1].

The new reference to needs assessment for musculoskeletal conditions is long overdue considering the size of the burden that these conditions present. For example, WHO estimates that 10% of the world's population aged 60 years and over have significant clinical problems that can be attributed to osteoarthritis (OA). It also identifies OA to be the fourth leading cause of disability by the year 2020 [3]. Knee OA is the most common type of OA in older people, and is characterised by the clinical syndrome of knee pain and related disability. However, many people who experience knee pain do not have a clinical diagnosis, either because they do not consult health professionals, or because no firm diagnosis was made.

Needs assessment requires identifying felt need (an individual assessment that there is a need for health care) and an investigation of whether felt needs are turned into an expressed need (demand for health care) [4]. The dominant decision-making framework for health needs assessment in current health care delivery is the Stevens and Gabbay model [5]. In this model gaps may occur between need (what people can benefit from), demand (what they ask for) and supply (what services are provided). Our previous work [6] has focused on identifying the burden of knee pain in the population and identifying patterns of health care use. However, the question of understanding the relationship between felt and expressed need (demand) requires reference to a broader socio-cultural interpretation of need and it is at this juncture that our paper is situated.

Our first aim was to examine knee pain and disability as reported by individuals participating in a population survey (an indication of felt need). Our second aim was to investigate subsequent health seeking-behaviour in order to understand the rationale behind peoples' decisions to seek or not seek health. This would enable any disjuncture between felt and expressed need to be explored and provide a model for understanding needs in relation to musculoskeletal pain, now a requirement of the MSF.

## Methods

Each phase of the study was approved by the North Staffordshire Research Ethics Committee.

We used a population survey as one method for identifying symptoms (and the potential need for care) in the community. The survey was also used as the sampling frame for the qualitative study. In March 2000 we mailed a postal questionnaire to all 8995 adults aged 50 and over who were registered with 3 general practices in North Staffordshire. The survey was about general health, knee pain and disability and related health care use, the results of which have been previously reported [6]. Of the original study population, 6792 people (77% adjusted) responded of whom 5784 were still registered at the practice in 2003 when we mailed a follow-up questionnaire, the results of which are presented in this paper. The questionnaire included the Knee Pain Screening Tool (KNEST) [6] which captures self reported data on the presence of knee pain and use of health care in the last 12 months. The knee pain screening question used in the KNEST is "Have you had pain in the last year in or around the knee?" [6]. The questionnaire also included demographic questions and for those who report knee pain, additional questions on treatments and medications used in the last two weeks and the Western Ontario and McMaster Universities Osteoarthritis Index (WOMAC) [7]. A question on the use of home remedies for knee pain in the last two weeks was derived from the Somerset and Avon Survey of Health (with permission).

The knee pain screening question (on the KNEST tool) was used to calculate the 12-month period prevalence of all knee pain. The WOMAC was used to provide data on symptom and functional severity. Item responses for the WOMAC were summed to produce subscale scores (pain range 0-20, stiffness 0-8, physical function 0-68) as recommended by the developers [7]. Higher scores indicate worse health. Recommended guidelines for dealing with missing data were also followed. As there is no agreed cut off to define severity of pain or disability in the WOMAC literature, severity of WOMAC items was defined categorically by grouping WOMAC responders who scored "severe" or "extreme" on at least one item on the pain or physical function scales into a "severe" group. This generates a distinct group of responders who share a perception that some aspect of their lives is severely restricted by pain or disability. The remainder were described as "non-severe". Descriptive statistics and mean scores were calculated for each WOMAC domain.

Whilst the survey was being administered we undertook 5 pilot interviews for the qualitative study. The sample for this was drawn from another survey that was being undertaken in a different general practice. The interview method for the main qualitative study did not change from the method used in the pilot study. Therefore, we have included the 5 pilot interviews in the analysis detailed below.

4317 people responded to the KNEST follow up survey and 58% of these gave consent to further contact. We stratified responders by 4 variables, whether they had consulted their GP in the last twelve months or not, chronicity of knee pain (chronic = more than three months, or non-chronic = less than three months), by age (over or under 75) and gender. We therefore ended up with 16 groups.

In order to gain a breadth of experiences, we wanted to interview at least one individual from each cell. We randomly sampled 4 responders to each cell from the database to account for non-participation rates. We wrote to the first 16 (one from each cell) inviting them to take part in a qualitative interview focusing on their experiences of knee pain and disability. We continued to mail invitations until at least one from each cell was recruited.

We invited the participants who were recruited from the KNEST survey (n = 17) to also participate in a diary study. Ten agreed to complete an open diary for 1 week. The unstructured format of the diary allowed for silenced accounts to be surfaced [9]. The qualitative study in this paper therefore comprises of 22 interviews (17 recruited from the KNEST survey and 5 pilot interviews) and 10 diaries. Data has been anonymised and the names given are pseudonyms.

The characteristics of all of the participants in the qualitative study (n = 22) are outlined in Table 1. All of the interviews were conducted by the same researcher. An interview guide was used and a semi-structured approach was adopted. The interviews were tape recorded and transcribed verbatim. The data was managed using the qualitative data software NVivo. Three researchers (CJ, PO, JR) independently coded two transcripts and a coding framework was derived. Each researcher then coded a proportion of the transcripts, and coding was checked by another researcher. NVivo was used to store memos and developing ideas about the project from all three researchers.

**Table 1:** Characteristics of participants in the qualitative study.

**Pseudonym**	**Gender**	**Age**	**Knee Status**	**Consulted GP for knee pain in last 12 months**
Robert	M	77	Non-chronic^a^, non-severe^b^	No
James	M	59	Chronic, severe	No
Jenny	F	66	Non-chronic, non-severe	Yes
Roy	M	80	Chronic, Severe	No
Fred	M	85	Non-chronic, non-severe	No
David	M	59	Chronic, severe	No
Susan	F	65	Non-chronic, non-severe	No
Steven	M	55	Chronic, non-severe	No
Heather	F	53	Non-chronic, severe	Yes
Peter	M	72	Non-chronic, severe	No
John	M	60	Non-chronic, non-severe	No
Joyce	F	60	Chronic, severe	Yes
Elizabeth	F	76	Non-chronic, non-severe	No
Mary	F	76	Chronic, non-severe	Yes
Geoff	M	79	Chronic, severe	No
Brenda	F	85	Chronic, non-severe	No
Colin	M	58	Non-chronic, non-severe	Yes
Barbara	F	61	Chronic, non-severe	No
Shirley	F	67	Chronic, non-severe	Yes
Stuart	M	54	Chronic, severe	No
Keith	M	83	Non-chronic, severe	Yes
Carol	F	76	Chronic, severe	Yes

^a^Chronicity of knee pain defined by KNEST screening question. Chronic pain is pain ≥ 3 months duration, non-chronic pain defined as pain <3 months duration.

^b^Responders who answered "severe" or "extreme" difficulty on at least one WOMAC Physical function item, or at least one item on WOMAC Pain item were classified as severe. All others classified as non-severe.

The interviews and diaries were analysed in two ways: first, all the material was coded focusing primarily on the views and interpretations of the participants, augmented by the themes and thoughts that researchers drew from the texts. The analysis was built from the themes that emerged from the coding and categorisation of data and was based upon the approach used by Strauss and Corbin [10]. Second, where interviews or diaries contained detailed stories, we used an analytical approach that was informed by narrative theories about the construction of illness stories [11]. The way in which the participants spoke or wrote about their experiences provided insights into their construction of self and the ways in which they conveyed this to others, including health care professionals. Maintaining the integrity of the narrative emphasised the logic and the social context.

In this paper we focus on the main issues that are pertinent to understanding the different aspects of need, in particular, felt need, health care use and the use of medications/self care. In our analysis we draw on the data collected from the epidemiological survey, interviews and diaries and present the findings in an integrated manner, according to how key themes are illuminated through the different methods.

The purpose of embedding the qualitative research within the quantitative survey was to recognise the knowledge derived from both approaches: quantitative surveys aim to generalise from representative samples to populations, while qualitative research arrives at idiographic generalisation based upon knowledge derived from and about cases [8]. The different kinds of generalisations are necessary to inform health policy and practice, where understanding need is central to both.

## Results

### Patterns and descriptions of need

**Figure 1:**
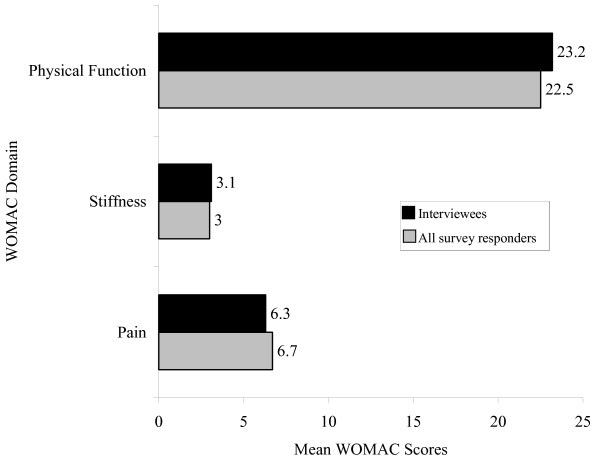
Mean WOMAC scores for all survey responders with knee pain compared to those who participated in the interviews.

Among responders, 4035 (93%) answered the KNEST knee pain question. The 12-month period prevalence of pain "in or around the knee" was 1999/4035 or 49.5% (95% Confidence Interval 48.0% – 51.1%). In this group of knee pain sufferers, half reported severe knee pain and disability. Mean WOMAC scores for all KNEST survey responders with knee pain and the interview sample are shown in Figure 1.

As might be expected the mean WOMAC scores from our population data are lower than those reported from other studies where patients have a diagnosis of osteoarthritis and are awaiting referral for physiotherapy or knee arthroplasty or attending hospitals clinics [12-14].

Felt needs can also be described by investigating individual WOMAC items. Twenty five percent of survey responders with knee pain reported severe or extreme pain going up or down stairs, 12% reported severe or extreme pain standing and 11% had severe or extreme pain walking. The activity causing the most difficulty with physical functioning was heavy domestic duties with 30% reporting severe or extreme problems. Twenty eight percent reported severe or extreme difficulty bending, 24% getting in and out of bath, 19% getting up stairs and 18% had severe or extreme difficulty getting in or out of a car. These data show the impact of pain and disability on everyday activities.

The interviews and diaries offered compelling fragments of people's experiences that resemble what Radley [15] defines as first-person accounts that display the author's pain, and thereby induce the reader to appreciate the world of his or her suffering by 'making it present again.' Most people talked about knee pain in relation to specific activities such as being still in one position for some time, going up and down stairs or walking. The qualitative data, therefore, underpins the survey results.

"I mean, if I sit too long, that doesn't help either. But the worst part is if I'm asleep and my legs are bent and I haven't woke up, the pain, I can't tell you what it is like. I can not move it...and what I do is I grip both hands round the knee and try to force my leg straight and I break out in a hot sweat. All I can say is that it is a bony pain. I could shout out with the pain." (Heather)

The level of pain ranged from what was described above as discomfort, to severe pain that stopped people from undertaking many of their normal daily activities.

"When it first happened [knee pain], I couldn't put weight on my foot. It was horrible. I can't tell you what it was like. Really really severe....painful; absolutely painful. I used to walk a lot, that stopped me from walking, but now I'm walking again so that's better isn't it?' I thought I'd be a cripple for life. I couldn't see it going. I couldn't see what would make it go, but physio helped and those tablets helped." (Susan)

In common with other musculoskeletal pain [16] many people explained that the pain was not a constant phenomenon, and that fluctuations occurred even though these could not always be explained. Coping with these ups and downs depended on a number of contextual factors such as social support, access to services and psychological well-being. The interaction of the latter two is illustrated by the following quotation:

"...if I'm 54 now, another 10 years, you know, am I going to be back to square one? Is it worth going through all that? It depends on how you feel: oh, yes, again, with me 'down' a bit. I'm going to go [to the doctor] and another time I say: Oh, I can cope with it." (Heather)

Sanders and colleagues [17] reported that older people with knee pain minimised their suffering because they accepted it as part of normal ageing. Our study reinforced this finding and the excerpt below from one of the diaries is representative of this approach:

"Had some pain and stiffness in my knees later in the day when squatting/stooping down for a short while looking in a low cupboard – pain was around the knee joint. This faded away when I stood up and flexed the joint – getting erect was a struggle. I find this frustrating at times, but accept it as one of the disadvantages of growing old." (Peter)

While pain was clearly a feature of everyday living for this person – and many others in our survey – redefining it as 'frustrating' and as an inevitable part of the ageing process meant that pain was 'downgraded', and consequently turning felt need into expressed need did not happen. Thus, when asked in the epidemiological survey, people did report knee pain, but the interviews and diaries explained that the existence of this pain was not necessarily acknowledged as a symptom of illness. The mediating influence of cultural concepts of ageing and interpretations of the thresholds for presenting to health professionals (i.e. fluctuating and intermittent pain did not warrant consultation) appeared to suppress the acknowledgement that the pain experience need not be borne.

### Health care use

Among responders with knee pain (according to the KNEST knee pain screening question), 33% (*n *= 630) reported visiting their GP about this in the last 12 months. In the group with severe pain or physical functioning over half had not consulted a GP in the last twelve months about their knee problem (53% had not consulted a GP). There was a considerable group of people with felt need who did not consult their GP in a 12-month period and might have potential to benefit from advice, treatment and preventive strategies. Furthermore, one in ten survey responders with severe knee pain or disability had not consulted a GP in the last 12 months about their knee problem and had not used medications (prescribed or over the counter), aids or self management (exercise, heat, cold, bandage or knee support, walking stick or creams or sprays) or home remedies for their knee pain in the last two weeks.

The interviews and diaries illuminate some of the reasons why people did or did not express their health care needs. Pain intensity, perceived high impact on daily life were the most cited reasons for consultation. For example:

"I think they must have been.. they must have been really painful then. I think that's why I went and, erm.. any way, she had.. they had a look at them, you know, and she sent me for an x-ray." (Jenny)

Very painful.. couldn't put weight on it. I couldn't put weight on it. I went to the doctor and he gave me some pain-killers and then I went to my son. He's got a practice in Crewe and had about 12 sessions of physio on it and even then it didn't 'go.' (Susan)

Pressure from family members also had an influence on the help seeking behaviour of some participants:

"So, obviously it was pressure from my wife.. we might as well say that, erm... it was the final push to go down, obviously to see him, 'cos it was getting no better. She said, 'look,' you know, 'you don't know what you've done,' or whatever.. you know, 'you need to go.' So, 'all right, I'll go then!' So.. off I go. ...and my daughter as well.. 'cos they kept working on me, didn't they, as well." (David)

During the interviews, more reasons were given for not consulting health care rather than for consulting, and this reflects findings of other studies [18]. Our study reinforces the contention that help-avoidance represented a complex phenomenon: expectations of treatment were shaped by ideas about effectiveness, by the threshold limit that needed to be reached before consultation was justified or by notions of being a deserving case – in comparison to others with more serious health problems. The most prominent sentiment expressed explaining help-avoidance contained negative judgements of effectiveness:

"I haven't been to the doctors about it because I can't see any point, they can't operate and all they'll say is we'll give you ...I mean, we've got some fine doctors, so no, there's a limit to what they can do. Well, I mean, ...I don't even go to the hospital now. I mean, it's just, ...I take it that there's nothing you can do about it. I ...all I go to see him is ...well, I don't really go for anything bar my ... six monthly check-up. No, I never say anything. As I say, there's not a lot of point. All he could do is give me another painkiller and that's it." (Roy)

The interpretation that there was a limit to what can be done about the knee pain appeared to be linked to an implicit notion of cure, symbolised by an operation. Within that context giving medication seemed to be viewed as inferior treatment, and almost dismissed by using the words "all he could do is give me another painkiller." Continuing consultation, therefore, was deemed to be of little value as no effective curative treatment could be offered.

Lack of effectiveness was reinforced when knee pain was linked to ageing, and in particularly, the notion of 'wear and tear' which was mentioned in consultations:

"I've been ...I've seen him...but all he said to me, you see (is), it's wear and tear. When he describes wear and tear if it's ...it's just age and it's just a 'whatsit'...as if nothing can be done for you [...] . With him telling me it was wear and tear that meant they couldn't do anything, but I don't know whether they can or not." (Geoff)

Clearly the perception that age, wear and tear and no effective treatment were inextricably linked meant that this person considered consultation futile. Many other participants talked about the concept of wear and tear and its negative impact on the thinking of health professionals, and in turn on their patients. The following excerpt made this very clear:

"Well, he [doctor] just took one look at it [knee] and just went: "live with it"." (John)

It may be that some GPs similarly view available interventions as having limited value, and a parallel can be drawn with studies that compare treatments for hip or knee problems, whereby referral for joint replacement is much lower for the knee than the hip [19].

Sociological studies have highlighted the issue of comparison, where people assess their own health status against others in their social network or with people suffering from similar conditions [20]. Moral judgements concerning one's status as deserving attention and health care were made within that comparative context, and even though individuals might suffer from a high degree of pain (as measured by the WOMAC) they did not consult:

"I don't think there is anything they can do really. Not going to give me two new knees, are they? I mean, and I wouldn't want two new knees, 'cos other people have much worse that need two new knees don't they?" (Barbara)

In contrast, a number of people gave explanations that appeared more pro-active, underlining their sense of self and in particular, their need for independence. One participant said in her interview that she was a 'very independent person' and this self image, combined with the notion of what constitutes a deserving case meant that she primarily managed her pain with prescribed or over the counter medicines. The other reason given for choosing not to consult was related to prioritising health problems. Many older people lived with more than one condition, and they often ranked them in terms of severity and perceived urgency:

"Well, they know I've got a bad knee, so there's no point in going down again, just for that. You know, if I needed her, yes, I would go. When they found the lump in my neck – Dr.W. was up here [...] and I had very quick treatment to check what it was. I was at the hospital and I'd seen a specialist within three days." (Shirley)

While the survey highlighted the fact that many people experienced moderate to severe knee pain, it also revealed that a large proportion did not consult their GP. The reasons for not consulting were explored in the interviews and diaries, providing evidence of clear choices made on the basis of judgements about effective interventions, threshold values, self-identity and prioritising health problems. An issue raised in earlier research [21] might be relevant here too, namely that by not consulting people felt that they maintained their status as healthy (i.e. not being a patient) and therefore enhanced their ability to cope with pain and disability. This highlights the limitations of using a single concept of health care need. When asked in the epidemiological survey people did report knee pain, but the interviews and diaries explained that the existence of this pain was not necessarily acknowledged as a symptom of illness.

**Figure 2:**
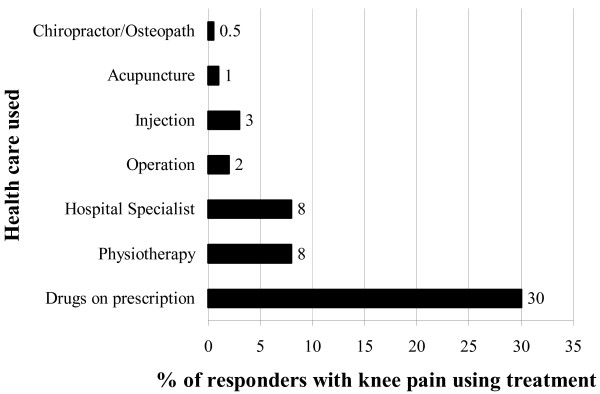
Use of NHS services for knee pain in the last 12 months by adults aged 50 and over living in the community.

Accessing health care other than the GP was reported in the survey, shown in figure 2 below.

Medicines use and self care featured prominently in the survey and qualitative study, and we discuss both types of use in more detail.

### Medicine use

The previous graph demonstrated the prominent role of medication, despite interviewees expressing doubts about its effectiveness. When asked in the survey specifically about medicines use in the last two weeks, 63% of responders had taken some medication. Paracetamol was most commonly cited with 28% of responders indicating they had used this medication. Other common painkillers were Ibuprofen (16%), Co-proxamol (15%), Co-codamol (12%), Aspirin (7%), Diclofenac (4%), Dihydrocodeine (2.6%) and Naproxen (1%). (Co-proxamol has since been withdrawn due to concerns over its safety).

The continued use of medication was explained by many participants within the context of coping with disabling pain, and thus pain intensity and duration were the main factors determining this. The 'social contract' that patients have with their GP shapes their view that it is legitimate for GPs to prescribe drugs and for patients to take them [22]. Taken together these factors were strong drivers for people to adopt medications use as a strategy for managing pain. At the same time, ambivalence about taking medicines featured as a dominant theme in the interviews, and this reflects the findings from other researchers who report reasons for aversion to medicines that include: fear of drug dependency, stoicism, maintaining normality and identity, differences in expectations between professionals and patients, and concerns about the safety of medicines [20,23-25]. The fear of side-effects was particularly prominent in relation to non-steroidal anti-inflammatories.

" [...] So she put me on a stronger Ibuprofen type of slow release which ...seems to help. She wanted me to have two a day, one in the morning and one at night, but I won't. I only have one in the morning. Sometimes I don't even have that cos like I say, I don't want to be stuck with tablets. I'm wary of side effects [...] You hear of the Ibuprofen type of thing can give you stomach bleed or anything. I don't want that, you know, or indigestions." (Shirley)

The level of discussion with the GP or other health professionals about the pros and cons of taking NSAIDs did not appear to be high, and in the interviews people said that they tended to make their own decisions about dosage. This reflected findings from other studies that people try to take as little medication as possible [20].

### Self care and home remedies

**Figure 3:**
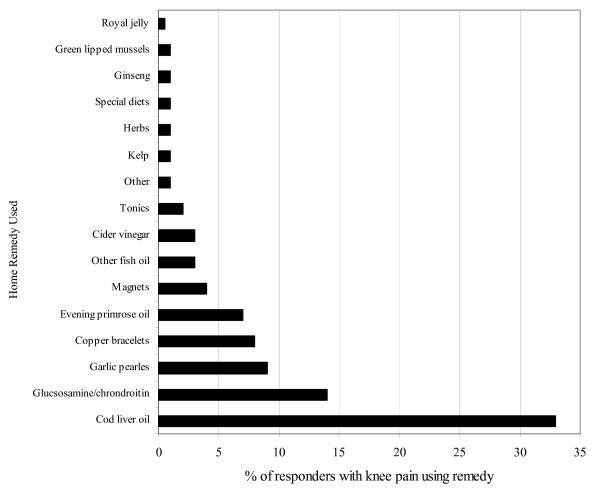
Use of home remedies for knee pain in the last two weeks by adults aged 50 and over living in the community.

The survey asked for use of complementary or alternative remedies and Figure 3 shows the extent of uptake. The most common remedy being used was cod liver oil with 33% of survey responders using this for their knee pain in the last two weeks. Glucosamine and Chondroitin were also common, with over 14% of responders with knee pain using this for their knee problem. These figures are similar to those reported by Jordan and colleagues [26] who studied adults aged 55 and over with a clinical diagnosis of knee OA.

The mechanisms that influence either a shift towards complementary and alternative medicine, or its use in parallel to conventional treatments are still not well understood and research on people with OA is just beginning [27]. Responders gave a further reason as to their decision to turn to other sources of help because they felt they had reached 'the end of the line':

"Everything that comes on the telly, I say...Oh, I'll try those, I'll try one of those, you know see how it works. Nothing really cures it but it does ease the pain." (Joyce)

While people did not elaborate in much detail on the use of alternatives, the diary study contained the account of one gentleman who described his strategies as follows:

07.30. My wife massaged my feet and legs, bathed them and applied cream.

19.00 – 19.25. Reflexology applied by my wife, felt completely relaxed afterwards. (Keith)

In between the two time periods he did exercises and tried to walk, but he obviously derived most benefit from the treatments administered by his wife. Rather than emphasising pain relief, he used the word 'relaxed'. This might be an indication that the more holistic approach of complementary therapies positively influenced his feelings about pain.

## Discussion

Musculoskeletal problems, and in particular, knee pain and associated disability are increasingly prevalent, yet, recognition of these problems remains patchy: people living with knee pain often downplay the impact on their daily life; health professionals do not always investigate, treat or provide advice to a sufficient degree; health policy makers do not accord musculoskeletal health the level of priority commensurate to the size of the problems. Given this context our population based survey identifying knee pain and disability (felt need) can be judged to be an important step towards a better understanding of the burden of musculoskeletal ill-health. The qualitative study offered insightful glimpses into the feelings and perceptions of living with and adapting to pain and the translation of felt need into expressed need for care.

Despite the considerable level of problems reported many people do not seek help from conventional or complementary health professionals. The explanation of the gap between felt need and expressed need (demand) in our study is complex, and echoes the findings of earlier studies [17,28] but also extends them. First, people appear to minimise their knee pain or characterise it as a normal part of ageing [17], thus accepting it as something they 'have to live with'. Second, the 'normalisation tendency' feeds the popular image of knee pain and osteoarthritis as 'wear and tear', emphasising its inevitable and incurable nature. If this is people's dominant belief it becomes logical that they do not seek help. Third, this perception tends to be reinforced if health professionals adopt a similar language of ageing and degeneration, and has as a corollary that people embrace the notion that 'there isn't anything that can be done'. Taken together, these three sets of factors represent a logical response that inhibits felt needs to being translated into expressed need. In addition, by adopting a mixed methods approach, we have also identified a tension between the conceptualisations of need. The survey revealed a high prevalence and impact of pain. This offers clear evidence that knee pain should be a public health priority. The diaries and interviews, however, revealed a personal dimension to assessment of need, which de-prioritised symptoms and the need for care. Our message is not the need to increase GP consultations *per se*. If older adults are not contacting health care services for their knee pain it does not necessarily represent an unmet need for care. It is possible that not seeking health care is a measure of successful self-management of pain. However, given the impact and severity of pain in those who choose not to seek care, we emphasise the missed opportunity for providing lifestyle and self-management advice.

## Conclusion

A broader socio-cultural interpretation of need is required in order to fully understand the relationship between felt and expressed need for musculoskeletal conditions. If the new musculoskeletal framework is to be implemented, health care planners and clinicians will need to identify musculoskeletal conditions as priority, draw, and act, upon a newly emerging range of evidence about effective interventions [29], but also actively engage with the older population to dispel the myth that "nothing can be done". The vision of the MSF framework is to enable access to high quality, timely advice, assessment and treatment to enable people with musculoskeletal conditions to fulfil their optimum health potential and remain independent [1]. An integrated and evidence based approach to needs assessment will enable a common understanding of the need for health care, and goes some way to helping this vision to be achieved.

Our research highlights that no one single approach or tool will be able to uncover the complexity of need, and that survey methodologies are usefully complemented by in-depth qualitative approaches. The challenge is to truly integrate the findings in order to compare the individual-level experiences with the pattern of need at the population level. Opportunities are opening up through the policy changes that are placing the patient's voice more centre-stage, and systematic collection and analysis of patient experiences need to be developed further. For example, representative and repeat focus group interviews, open-ended questions to be included in patient surveys and regular studies as the one reported in our paper are viable options to investigate health needs from individual, sub-group and population perspectives. Such studies which focus on a more holistic understanding of need will be better able to identify the nature and extent of unmet need, and provide guidance for targeting health care interventions.

## Competing interests

All authors are independent of the funders. The authors declare that they have no competing interests.

## Authors' contributions

All authors contributed to this manuscript. CJ conceived the design of the study and coordinated the study. JR, BNO and CJ all undertook analysis and participated in drafting the manuscript and in interpreting the data. All authors read and approved the final manuscript.

## Pre-publication history

The pre-publication history for this paper can be accessed here:


